# Monkey CV1 cell line expressing the sheep–goat SLAM protein: A highly sensitive cell line for the isolation of peste des petits ruminants virus from pathological specimens

**DOI:** 10.1016/j.jviromet.2011.02.024

**Published:** 2011-05

**Authors:** Caroline Mélanie Adombi, Mamadou Lelenta, Charles Euloge Lamien, David Shamaki, Yao Mathurin Koffi, Abdallah Traoré, Roland Silber, Emmanuel Couacy-Hymann, Sanne Charles Bodjo, Joseph A. Djaman, Antony George Luckins, Adama Diallo

**Affiliations:** a Animal Production and Health Laboratory, FAO/IAEA Agriculture & Biotechnology Laboratory, IAEA Laboratories Seibersdorf, International Atomic Energy Agency (IAEA), Wagramer Strasse 5, P.O. Box 100, A1400 Vienna, Austria; b National Veterinary Research Institute, P.M.B. 01, Vom, Plateau State 930001, Nigeria; c Laboratoire Central de Pathologie Animale, BP 206, Bingerville, Côte d’Ivoire; d Laboratoire Central Vétérinaire, BP 2295, Bamako, Mali; e Institute for Veterinary Disease Control, Austrian Agency for Health and Food Safety, Robert Koch-Gasse 17, A-2340 Moedling, Austria; f UFR Biosciences (Université de Cocody) et Département de Biochimie, Institut Pasteur de Côte d’Ivoire, 01 BP 490, Abidjan 01, Université d’Abidjan, Côte d’Ivoire

**Keywords:** Morbillivirus, Paramyxoviridae, PPRV, SLAM, Immunosuppression, Lymphopaenia

## Abstract

Peste des petits ruminants (PPR) is an important economically transboundary disease of sheep and goats caused by a virus which belongs to the genus *Morbillivirus*. This genus, in the family *Paramyxoviridae*, also includes the measles virus (MV), canine distemper virus (CDV), rinderpest virus (RPV), and marine mammal viruses. One of the main features of these viruses is the severe transient lymphopaenia and immunosuppression they induce in their respective hosts, thereby favouring secondary bacterial and parasitic infections. This lymphopaenia is probably accounted for by the fact that lymphoid cells are the main targets of the morbilliviruses. In early 2000, it was demonstrated that a transmembrane glycoprotein of the immunoglobulin superfamily which is present on the surface of lymphoid cells, the signalling lymphocyte activation molecule (SLAM), is used as cellular receptor by MV, CDV and RPV. Wild-type strains of these viruses can be isolated and propagated efficiently in non-lymphoid cells expressing this protein. The present study has demonstrated that monkey CV1 cells expressing goat SLAM are also highly efficient for isolating PPRV from pathological samples. This finding suggests that SLAM, as is in the case for MV, CDV and RPV, is also a receptor for PPRV.

## 1 Introduction

Peste des petits ruminants (PPR) is a highly contagious viral disease of sheep, goats and wild small ruminants (Abu Elzein et al., 2004; Furley et al., 1987; Lefèvre and Diallo, 1990). PPR is a transboundary animal disease causing significant economic losses due to its high morbidity and mortality, and has therefore been classified among diseases that must be notified to the World Organisation for Animal Health (OIE). It poses a serious threat to the development of small ruminant production in all areas where it occurs, namely Africa, the Middle East and Asia. As poor people in developing countries rely on small ruminants, particularly goats, for their livelihoods, it has been suggested that the control of PPR in endemic regions should be reflected in developing poverty alleviation policies (Perry et al., 2002). Clinically, PPR is characterized by pyrexia, necrotic stomatitis, catarrhal inflammation of the ocular and nasal mucosae, bronchopneumonia, diarrhoea and, in many cases, death. Apart from the bronchopneumonia, all these clinical signs resemble those of rinderpest, the cattle plague. The causal agents of both diseases are closely related viruses, peste des petits ruminants virus (PPRV) and rinderpest virus (RPV). They are classified in the genus *Morbillivirus* within the family *Paramyxoviridae* (Gibbs et al., 1979). Other members of this genus include measles virus (MV), a serious human pathogen, canine distemper virus (CDV) affecting animals of the family *Canidae*, phocine distemper virus (PDV) and cetacean morbilliviruses (CMV) which affect marine mammals. All these viruses induce in their respective hosts a transient but severe immunosuppression which favours secondary opportunistic bacterial and parasitic infections responsible for many of the disease symptoms and the severity of the infection (Obi et al., 1983; Seki et al., 2003; Ugochukwu and Agwu, 1991). It is likely that several mechanisms contribute to this morbillivirus-induced immunosuppression including leucopaenia, a major sign observed during infection (Heaney et al., 2002; Rajak et al., 2005; Schneider-Schaulies et al., 2001) since lymphoid cells are a major target of morbilliviruses (Takeda et al., 2007; Wohlsein et al., 1993, 1995). This tropism is linked to the presence of a protein receptor on the cell surface, the signalling lymphocyte activation molecule (SLAM) also known as CD150 which is used preferentially by wild-type morbilliviruses to bind to the host (Baron, 2005; Cocks et al., 1995; Kruse et al., 2001; McQuaid & Cosby, 2002; Ono et al., 2001; Sidorenko and Clark, 1993; Tatsuo et al., 2000, 2001; Yanagi et al., 2006). However, although lymphoid tissues are major sites of morbillivirus replication, it is also observed that they can infect and replicate in epithelial cells of other organs such as the alimentary track epithelial cells; lung and kidney cells by utilising other types of cell receptors that have yet to be clearly identified (Hashimoto et al., 2002; Leonard et al., 2008; Plowright, 1964; Tahara et al., 2008; Takeuchi et al., 2003; Wohlsein et al., 1993, 1995). The infection efficiency for those cells is up to 100–1000 times less than that of the lymphoid cells (Hashimoto et al., 2002; Takeda et al., 2007), but because they are easy to maintain in culture *in vitro*, they have been favoured preferentially for morbillivirus isolation. In the case of PPRV, primary cultures of bovine kidney, goat kidney, sheep kidney and lung cells have all been utilised for isolation and maintenance (Abu Elzein et al., 1990; El Hag Ali and Taylor, 1984; Lefèvre and Diallo, 1990; Taylor et al., 1990; Taylor and Abegunde, 1979). However not only is the availability of primary cells becoming more problematic their quality is not guaranteed and there is considerable variation from batch to batch. These drawbacks in the use of primary cell cultures have stimulated the use of cell lines for PPRV isolation, in particular the African green monkey kidney (Vero) cell line (Lefèvre and Diallo, 1990). Unfortunately, as with other morbilliviruses, PPRV isolation using these cells is inefficient: the likelihood of isolating the virus is very low and even if successful it often requires multiple, sequential blind passages and many weeks in culture before the development of any cytopathic effect (CPE) can be observed (Abu Elzein et al., 1990; Albayrak and Alkan, 2009; Gulyaz and Ozkul, 2005; Lefèvre and Diallo, 1990).

Following the identification of the SLAM as the main receptor used by MV, CDV and RPV wild type strains (Baron, 2005; Hsu et al., 2001; Ono et al., 2001; Tatsuo et al., 2000, 2001), Vero or CHO cells expressing human, dog or bovine SLAM proteins have been used as a highly efficient means of isolation and propagation of these morbilliviruses (Ono et al., 2001; Seki et al., 2003; Tatsuo et al., 2001). This paper reports the development of a CV1 cell line stably expressing the goat SLAM protein. This new cell line, designated CHS-20, is highly efficient for isolating wild type PPRV from pathological specimens.

## 2 Materials and methods

### 2.1 Cell lines and culture

Flp-In-CV-1 cells, referred to as CV1, were purchased from Invitrogen (Eugene, Oregon, USA). They were grown in the Dulbecco's Modified Eagle Medium (DMEM) supplemented with 10% irradiated foetal calf serum (FCS) and 1% mixed antibiotic–antimycotic solution (Invitrogen, Eugene, Oregon, USA), 2 mM l-glutamine, 1 mM sodium pyruvate, and 100 μg/ml zeocin. The African green monkey kidney cells (Vero) were grown in DMEM medium containing 10% FCS and 1% mixed antibiotic–antimycotic solution (Invitrogen, Eugene, Oregon, USA).

### 2.2 Goat SLAM cDNA cloning

A cDNA sequence corresponding to open reading frame of the goat SLAM that was available in Genbank (Accession No. DQ228869) was synthesized and cloned into pUC57 vector by GenScript (Piscataway, NJ, USA). The cDNA was removed from the plasmid and sub-cloned into pcDNA5/FRT plasmid (Invitrogen, Eugene, Oregon, USA). The new plasmid, pFRT-CHS-20, was amplified in *Escherichia coli* DH5alpha bacteria, purified with the Endo-Free Plasmid Maxi Kit (Qiagen, Hilden, Germany) and used to transfect the cells.

### 2.3 Cells stably expressing goat SLAM

The pFRT-CHS-20 plasmid was used to transfect the CV1cells of the Flp-In system according to the manufacturer's instructions (Invitrogen, Eugene, Oregon, USA). Cells which had incorporated the recombinant plasmid were selected in a medium composed of the DMEM (Invitrogen, Eugene, Oregon, USA) supplemented with 10% FCS and the antibiotic hygromycin at a final concentration of 600 μg/ml. The cell clones which were resistant to this antibiotic were picked up and expanded in the selecting medium. The cloned cells were each cultured individually in a 25 cm^2^ flask and after 10 passages, total RNA was extracted for each clone using the RNAeasy mini kit (Qiagen, Hilden, Germany). From this total RNA, mRNAs were purified using the Oligotex mRNA kit (Qiagen, Hilden, Germany) and submitted to RT-PCR amplification using the following goat SLAM primers: Slam-BCO273F (5′aagagcaggaaggaggatgaagg3′) and Slam-BCO273R (5′gccaagagtgagatacaagaggtg3′). Briefly, 8 μl of each RNA was reverse transcribed (RT) into cDNA using the First Strand cDNA Synthesis kit (GE Healthcare, Buckinghamshire, UK) according to the manufacturer's instructions and using the random primers pdN (6). Five μl of the cDNA was obtained were used for the detection of goat SLAM mRNA by PCR with the goat SLAM primers indicated above. As a control for the quality of the extracted mRNA, a further five μl of the same cDNA was submitted to PCR to amplify mRNA corresponding to the β-actin gene by using the specific primers Bact1 (5′accaactgggacgacatggaga3′) and Bact2 (5′agccatctcctgctcgaagtc3′).

### 2.4 Viruses and clinical specimens

In this study, the live attenuated PPRV Nigeria 75/1 vaccine strain was used. This virus was isolated originally from a sick goat on primary lamb kidney cell culture in Nigeria and attenuated by serial passages on Vero cells (Diallo et al., 1989; Taylor and Abegunde, 1979). In addition, clinical specimens from sick sheep and goats with suspected PPR were collected in 2008 and 2009 from different locations in Nigeria and Côte d’Ivoire respectively. The details of the samples are summarized in Table 1.

### 2.5 PPRV identification by RT-PCR and quantitative RT-PCR (RT-qPCR)

Before attempting isolation of the PPRV from the suspected infected pathological specimens used in this study, they were first screened by the reverse transcriptase polymerase chain reaction (RT-PCR) assay for the detection of viral nucleic acid. An RNeasy mini kit (Qiagen, Hilden, Germany) was used to extract total RNA from these specimens. RNAs were also extracted from cell culture medium to detect the presence of virus by RT-PCR.

These extracted RNAs were then submitted to the amplification of the PPRV RNA by the classical RT-PCR as described previously by Couacy-Hymann et al. (2002). The amplified cDNA samples were analysed by electrophoresis in 1.5% agarose gel stained with ethidium bromide at a concentration of 1 μg/ml.

The RNA samples that were extracted from the pathological samples were analysed by the one-step reverse transcription quantitative real-time PCR (RT-qPCR) using the iScript One-Step RT-PCR Kit (BioRad, Hercules, CA, USA) The primers and probe, specific to PPRV nucleocapsid protein gene, were designed using the Allele ID 6 (Premier Biosoft International, Palo Alto, CA, USA). The primers PPRN-MGBTaqF1 (5′-ggactgggcctcgacagg-3′) and PPRN-MGBTaqR1 (5′-ggatcgcagctttgacttcttc-3′) were used in combination with the minor groove binder (MGB) Taqman probe PPRN-MGBTaq01 (FAM-5′tccttcctccagcataa3′-BHQ1). The reaction mixture, in a total volume of 20 μl, was made with 10 μl of the 2× RT-PCR reaction mix for probes, 400 nM of each primer, 200 nM of the probe and 0.4 μl of the iScript reverse transcriptase for one-Step RT-PCR, 2 μl of template. It was performed in hard shell PCR plates, 96-well WHT/CLR covered with the microseal “B” film (BioRad, Hercules, CA, USA). The samples were tested in duplicate and the reaction was performed using a CFX96 Real-Time PCR Detection System (BioRad, Hercules, CA, USA) under the following conditions: 50 °C for 10 min; 95 °C for 5 min and 40 cycles of 95 °C for 10 s and 60 °C for 30 s.

### 2.6 Virus isolation

Before using the CHS-20 cells to isolate PPRV from the suspected PPR infected pathological samples, they were first tested with the live attenuated PPRV Nigeria 75/1 vaccine strain. The cells were infected in a 25 cm^2^ tissue culture flask with 0.5 ml of a virus suspension diluted to produce a titre of 2 × 10^4^ TCID_50_/ml. As controls, Vero and CV1 cells were also infected in a similar way to the CHS-20 cells. The virus was allowed to adsorb on the cells at 37 °C for one hour with gentle shaking every 15 min. After this time, the flask was filled with 5 ml of the complete cell culture medium and returned to the incubator at 37 °C. It was examined daily by phase contrast microscopy.

The CHS-20 cells were compared with the Vero and CV1cells to determine their efficiency in the isolation of PPRV from the pathological tissue samples. The samples were minced and ground with sterile sand using a mortar and pestle to give a 10% suspension in serum-free DMEM media. The suspensions were clarified by low speed centrifugation. For virus isolation, 0.5 ml of the sample homogenate was allowed to adsorb for one hour at 37 °C onto the cell monolayer at 80% of confluence in 25 cm^2^ tissue culture flasks with shaking every 15 min for one hour. After this time, 5 ml of cell growth medium supplemented with 2% antibiotics was added to the flask and it was returned to the incubator. One day after infection, the cell supernatant was removed and replaced by 5 ml of fresh growth medium containing 2% antibiotics. The cells were examined daily by microscopic observation for the detection of the virus CPE. The cell culture medium was changed every 2 days, the concentration of the antibiotics being reduced to 1% from day 4 onwards. At each medium change, an aliquot of 200 μl was kept for RNA extraction and detection of PPRV specific nucleic acid by classical RT-PCR. Cells in flasks where no CPE was detected after 7 days of incubation were trypsinised for a blind passage. An RNA extract was prepared from an aliquot of these trypsinised cells for use in an RT-PCR to detect PPRV specific nucleic acid. This cell blind passage was repeated every week on 3 more occasions. The virus isolation was considered negative if no CPE was detected after the fourth blind passage. However in 2 cases, up to 12 blind passages were made.

## 3 Results

### 3.1 Establishment of a CV-1 cell clone stably expressing sheep–goat SLAM

**Fig. 1:**
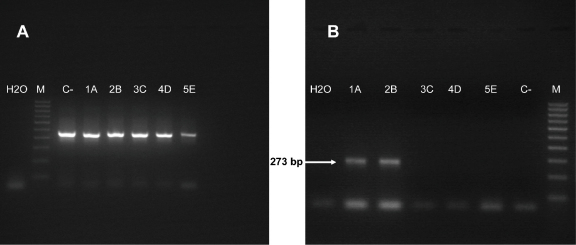
Photographs of the RT-PCR results to identify the cell clones potentially expressing the goat SLAM: (A) amplicons corresponding to the RT-PCR of the β-actin mRNA; (B) amplicons corresponding to the RT-PCR of the goat SLAM mRNA. Lane M, 100 bp DNA molecular weight ladder; lane C−, control cells non-transfected with the SLAM plasmid; lane 1A, transfected selected clone 1; lane 2B, transfected selected clone 2; lane 3C, transfected selected clone 3; lane 4D, transfected selected clone 4; lane 5E, transfected selected clone 5.

The pFRT-CHS-20 construct which comprises the pcDNA5/FRT plasmid into which was cloned the goat SLAM cDNA was used to transfect the CV1 cells as described in Section 2. Five cell clones, resistant to the selective antibiotic, were identified, picked up and expanded. In a subsequent step to characterize these cells, an immunofluorescence assay was performed using the mAb IPO-3 anti-human SLAM but it failed to detect expression of the goat SLAM protein (result not shown). This mAb, which recognizes the human SLAM, does not react with bovine or canine SLAM (Tatsuo et al., 2001). In order to test whether or not the selected cells were potentially producing the goat SLAM, another approach was used: the detection of mRNA corresponding to the transcription of the goat SLAM gene. In this case, mRNAs were purified from the selected antibiotic resistant cells and submitted to the RT-PCR using the goat SLAM primers. Another amplification was done from the same mRNA samples using primers specific to the gene of a ubiquitous protein, β-actin, to evaluate the quality of the purified extracted mRNA. As shown in Fig. 1, while all five clones were positive for the β-actin mRNA (Fig. 1A), only 2 were positive for the presence of the goat SLAM mRNA (Fig. 1B). A PCR test performed on an aliquot of the extracted mRNA without the RT step remained negative (result not shown), proof that the SLAM positive result from the two clones was not due to contamination from the plasmid used to transfect the cells nor from the cell genomic DNA. One of the cell clones which contained the goat SLAM mRNA, CHS-20, was selected and used for the rest of the study.

### 3.2 Identification of PPRV in pathological samples by classical and quantitative RT-PCR

Fifty six and 12 PPR suspected pathological tissue samples that were collected in 2008 and 2009 respectively in Nigeria and Côte d’Ivoire (Table 1) were analysed by both classical and quantitative RT-PCR using specific PPRV primers targeting the virus nucleocapsid protein (Np) gene. Twenty three of the 68 samples that were analysed were positive by the classical RT-PCR assay while the quantitative RT-PCR detected 3 more positive samples, 2 from lymph nodes and 1 from spleen. The results are summarized in Table 2. All 26 samples that tested PPR positive by these assays were submitted to the PPRV isolation trial on cell culture.

### 3.3 Use of CV1 cells expressing the goat SLAM protein, CHS-20 cells, to isolate PPRV from positive pathological samples

**Fig. 2:**
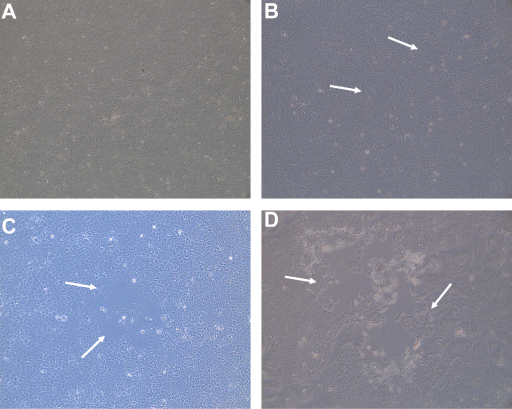
Photographs of the microscopic observation of the CHS-20 cells: (A) non infected cells, (B) cells infected with the attenuated PPRV Nigeria 75/1 (1 day post infection), (C) cells infected with the pathological specimen NIG/08-43 (2 days post infection), and (D) cells infected with the pathological sample CIV/09-01P (2 days post infection). Some of the syncytia are shown by the arrows.

Before using the CHS-20 cells to isolate PPRV from the pathological samples, they were first tested with the live vaccine PPRV Nigeria 75/1 strain which has been attenuated by serial passages on Vero cells (Diallo et al., 1989). One day after infection, syncytia were detected in many places in the flask containing the infected CHS-20 cells (Fig. 2B) but no modification was observed in the non-infected control CHS-20 cells (Fig. 2A). By day 2 the syncytia had enlarged so much that many of them had fused. By day 3 many cells had detached from the layer. The flask was then frozen and stored at −80 °C. In the Vero and CV1 cells, the CPE was slightly discernible only at day 3 post infection. It was characterized by the appearance of rounded, refringent cells that were clumped into grape-like clusters (not shown). They became detached from the cell layer without showing giant cells. At day 7 post infection, the CPE was more advanced and those flasks were also stored at −80 °C in the deep freezer, as with the previous flask.

**Fig. 3:**
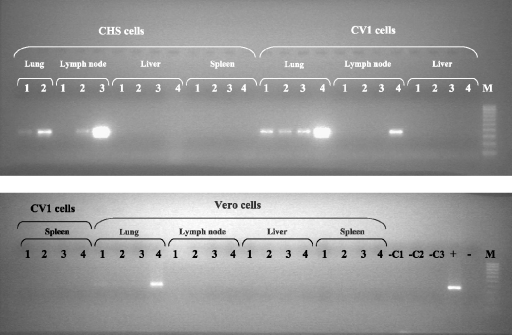
Monitoring the efficiency of CHS-20, CV1 and Vero cell lines in isolating PPRV from pathological specimens (lung, lymph node, spleen and liver) collected from the same animal: gel electrophoresis photograph of DNA amplified by RT-PCR of RNA extracted from different samples: 1 =  supernatant at day 2 post infection; 2 = supernatant at day 4 post infection; 3 = supernatant at day 8 post infection; 4 = cells collected after trypsination at day 8 post infection; − = negative control; + = positive control; −C1 = non-infected CHS20 cells; −C2 = non-infected CV1 cells; −C3 = non-infected Vero cells.

Following this preliminary test which demonstrated that the Vero cell-adapted PPRV vaccine Nigeria 75/1 strain caused the formation of syncytia in the CHS-20 cells but not in the Vero or the CV1cells, the efficacy of the SLAM-modified cells for isolating PPRV from the 26 pathological specimens that had been identified PPRV positive by the nucleic acid amplification assays was assessed (Table 2). The CV1 and Vero cells were also included in the isolation trial. In CHS-20 cell culture a CPE was detected from day 1 to day 10 after infection and without any subsequent blind passage. In a similar manner to the effects seen with the live vaccine strain, it was characterized by the appearance of vacuolated syncytia (Fig. 2C and D) in the cell monolayer while no CPE was observed in the control cells (Fig. 2A). The syncytia increased in size in 2–3 days to form large cell clumps that detached from the cell layer. The flask was frozen when the CPE covered about 70–80% of the cell layer. Out of the 26 samples that were used in this trial, the virus isolation on CHS-20 cells was successful with 16. In the case of the CV1 and Vero cells, no CPE could be detected after 4 blind passages. The cell culture supernatants were collected regularly and tested by RT-PCR to detect the eventual release of virus in the medium as indicated in Section 2. An aliquot of the cells was also collected at the time of the blind passage and tested for the presence of the PPRV RNA. While the release of the virus in the infected cell culture supernatant was effectively demonstrated for the 16 samples which had developed syncytia in the CHS-20 cells, virus could not be detected in the cell culture supernatant medium for the 10 remaining samples that did not develop CPE (data not shown). Fig. 3 details the efficiency of the three cells lines (Vero, CV1 and CHS-20) in isolating PPRV from lung, lymph node, spleen and liver collected from the same animal from Côte d’Ivoire. As of days 2 and 4, PPRV RNA is detected effectively in the supernatant of CHS-20 cells infected with the lung (CIV/09-01P) and lymph node (CIV/09-01G) specimens respectively (supernatants from day 1 were not tested). The CPE was visible in these cells at days 1 and 2 respectively (Table 2). In contrast, in the CHS-20 cells that were infected with specimens from the liver and the spleen, all samples remained negative by RT-PCR and no CPE could be detected. For the other cell types, PPRV RNA could be clearly detected in all samples, supernatants and cells, collected from only CV1 cells infected with the lung specimen (CV1/09-01P). For CV1 and Vero cells infected with CIV/09-01G and CIV/09-01P respectively, only the collected cell samples were clearly positive by RT-PCR. All other samples were negative. No CPE was seen in the infected CV1 and Vero cells that were positive by RT-PCR at the time of sample collection. The results obtained for all the samples that were tested are summarized in Table 2. In the CHS-20 cells, the CPE was detected at days 1 and 2 post infection for the lung and the lymph node specimen respectively. Since Vero cells are often used for PPRV isolation, those cells that were infected with the lung samples CIV/09-01P and NIG/08-43, and in which the virus was detected (Table 2), were submitted to more weekly blind passages. It was necessary to continue to sub-culture the cells for 11 and 12 weeks post-infection in order to detect CPE in the Vero cells infected with the CIV/09-01P and NIG/08-43 (not shown) samples respectively. The CPE was characterized by the appearance of rounded, refringent cells that were clumped into grape-like clusters. They detached from the cell layer without showing clear syncytia. After the first day of the appearance of the CPE, the cell culture medium was collected every 2 days and the final collection was done a week later.

## 4 Discussion

In this study a new cell line, CHS-20, was developed by inserting into the genome of the monkey cell CV1, cDNA corresponding to the goat SLAM protein gene. Since no anti-goat SLAM antibody was available to demonstrate the production of this protein in CHS-20 cells, the RT-PCR assay was used for amplifying the mRNA corresponding to the transcript of this protein gene. The infection of these cells by the live attenuated vaccine PPRV Nigeria 75/1 resulted in the development of detectable syncytia. This viral strain has been adapted to grow in Vero cells in which it causes a CPE characterized by the presence of rounded refringent cells. In these infected cells, very small syncytia are usually seen only after the eosin–haemalun dye-staining (Diallo, 2010; Diallo et al., 1989). In this study, infection of the CV1 cells produced a CPE identical to that seen on unstained, infected Vero cells. However, in CHS-20 cells, the CV1 cell line that contains the goat SLAM gene, this attenuated PPRV caused the development of large syncytia, like those obtained in cells expressing human, dog or monkey SLAM infected by MV, CDV or RPV (Erlenhoefer et al., 2001; Kobune et al., 1990, 1991; Ono et al., 2001; Tatsuo et al., 2001; Seki et al., 2003; Baron, 2005). Based on this result, and since the mRNA corresponding to the transcript of the goat SLAM gene was detected in the CHS-20 cells, it is concluded that these cells are certainly expressing the goat SLAM and that this protein could be used as a receptor by the Vero cell-adapted PPRV Nigeria 75/1. Previous studies carried out with MV and RPV demonstrated that the SLAM protein is not only the main cell receptor for morbilliviruses wild-type strains but can also be used by the cell-adapted MV and RPV viruses for which other receptors have been identified, namely the ubiquitous protein CD46 protein and heparin sulphate respectively (Baron, 2005; Naniche et al., 1993; Ono et al., 2001; Seki et al., 2003). Following the encouraging results obtained from infecting CHS-20 cells with the attenuated PPRV Nigeria 75/1 strain, the new cells were further evaluated for their capacity to enable PPRV isolation from pathological specimens containing wild-type viruses. For this evaluation, the 26 samples from small ruminant in which PPRV RNA had been detected by classical RT-PCR and RT-qPCR were used. From many of these samples PPRV was recovered in these CHS-20 cells without any blind passage. The CPE, characterized by the appearance of large syncytia in the cell layer, was detected for some samples and as early as one day post-infection in one occasion. However, with the same samples a CPE related to a virus infection could not be detected in CV1 and Vero cells even after 4 blind passages. The excretion of the virus into culture medium was detected by RT-PCR in the absence of a CPE in one instance in a Vero cells, and in two instances in the CV1 cells (Table 2). In this study as shown in Fig. 3, the CV1 cells seem to be more sensitive than the Vero cells for the isolation of PPRV from pathological specimens. However, and since Vero cells are used commonly for PPRV isolation and growth, the blind passages were continued for 2 lung sample-infected Vero cells. In these cases the infected Vero cells showed a CPE only after 11 and 12 weeks post-infection. The same samples produced syncytia on the CHS-20 cells within 1 and 2 days after infection. In order to cause a visible CPE requires the presence of adapted mutant (s) in the virus suspension which was (or were) obtained in sufficient quantities for the two samples only after 11 and 12 weeks of continuous culture. In the case of CHS-20 cells, this adaptation, probably to a receptor to enter into the host cell, was unnecessary. Based on the results of this study it is concluded that in addition to MV, CDV and RPV, another morbillivirus, PPRV, also uses the SLAM protein as a receptor to infect the host cell. However, as suggested for MV, SLAM is certainly not the only protein that can be used as a receptor by the PPRV wild-type strains (Tahara et al., 2008; Takeda et al., 2007; Takeuchi et al., 2003). Indeed, as in the case of MV and other morbilliviruses, histopathological studies carried out on different tissues collected from hosts infected with PPR has revealed the presence of the virus, as demonstrated by the detection of syncytia, in cells such as renal, hepatic, respiratory and alimentary tract cells which do not express the SLAM protein (Aruni et al., 1998; Kul et al., 2007). In these cells, wild-type PPRV strains might use an alternative receptor(s), probably less efficient than the SLAM protein, for binding the virus.

## 5 Conclusion

The integration of cDNA corresponding to the gene of the goat SLAM into the CV1 monkey cell genome resulted in a cell which expressed this protein. Infection of this cell by PPRV resulted in the development of syncytia in a similar manner to that demonstrated by Vero cells expressing human, canine or bovine SLAM protein and infected by MV, CDV of RPV. Preliminary tests carried out with this new cell line, CHS-20, have demonstrated its high sensitivity for isolating PPRV from pathological samples. This finding will constitute an important element in the future for the diagnosis of PPR by facilitating the isolation of PPRV from pathological specimens.

## Figures and Tables

**Table 1:** Detail of the 68 clinical tissue specimens used in this study: 56 samples were collected in 2008 in Nigeria and 12 were collected in Côte d’Ivoire in 2009.

Sample ID	Tissue	Animal species	Month/year	Location	Country
NIG/08-01	Lymph node	Caprine	Unknown	Kaduna	Nigeria
NIG/08-02	Spleen	Caprine	Unknown	Kaduna	Nigeria
NIG/08-03	Lung	Caprine	Unknown	Kaduna	Nigeria
NIG/08-04	Lymph node	Caprine	Unknown	Kaduna	Nigeria
NIG/08-05	Spleen	Caprine	Unknown	Kaduna	Nigeria
NIG/08-06	Lung	Caprine	Unknown	Kaduna	Nigeria
NIG/08-07	Spleen	Caprine	Unknown	Kaduna	Nigeria
NIG/08-08	Lung	Caprine	Unknown	Kaduna	Nigeria
NIG/08-09	Lung	Caprine	5/08	Nsukka	Nigeria
NIG/08-10	Lymph node	Ovine	5/08	Nsukka	Nigeria
NIG/08-11	Spleen	Ovine	5/08	Nsukka	Nigeria
NIG/08-12	Lung	Ovine	5/08	Nsukka	Nigeria
NIG/08-13	Liver	Ovine	5/08	Nsukka	Nigeria
NIG/08-14	Lymph node	Caprine	5/08	Nsukka	Nigeria
NIG/08-15	Spleen	Caprine	5/08	Nsukka	Nigeria
NIG/08-16	Lung	Caprine	6/08	Jos	Nigeria
NIG/08-17	Lung	Caprine	8/08	Eket	Nigeria
NIG/08-18	Spleen	Caprine	8/08	Eket	Nigeria
NIG/08-19	Lung	Caprine	5/08	Nsukka	Nigeria
NIG/08-20	Liver	Caprine	5/08	Nsukka	Nigeria
NIG/08-21	Spleen	Caprine	5/08	Nsukka	Nigeria
NIG/08-22	Spleen	Caprine	6/08	Jos	Nigeria
NIG/08-23	Liver	Caprine	6/08	Jos	Nigeria
NIG/08-24	Lung	Caprine	9/08	Misau	Nigeria
NIG/08-25	Spleen	Caprine	9/08	Misau	Nigeria
NIG/08-26	Lymph node	Caprine	9/08	Misau	Nigeria
NIG/08-27	Lung	Caprine	5/08	Zaria	Nigeria
NIG/08-28	Liver	Caprine	5/08	Zaria	Nigeria
NIG/08-29	Spleen	Caprine	5/08	Zaria	Nigeria
NIG/08-30	Lymph node	Caprine	5/08	Zaria	Nigeria
NIG/08-31	Lymph node	Ovine	7/08	Vom	Nigeria
NIG/08-32	Lymph node	Ovine	7/08	Vom	Nigeria
NIG/08-33	Trachea	Ovine	7/08	Vom	Nigeria
NIG/08-34	Lung	Ovine	7/08	Vom	Nigeria
NIG/08-35	Lung	Ovine	7/08	Vom	Nigeria
NIG/08-36	Trachea	Ovine	7/08	Vom	Nigeria
NIG/08-37	Lung	Caprine	6/08	Jos	Nigeria
NIG/08-38	Spleen	Caprine	6/08	Jos	Nigeria
NIG/08-39	Lung	Caprine	6/08	Ilesha	Nigeria
NIG/08-40	Spleen	Caprine	6/08	Ilesha	Nigeria
NIG/08-41	Spleen	Caprine	8/08	Azare	Nigeria
NIG/08-42	Lung	Caprine	8/08	Azare	Nigeria
NIG/08-43	Lung	Caprine	5/08	Zaria	Nigeria
NIG/08-44	Liver	Caprine	5/08	Zaria	Nigeria
NIG/08-45	Spleen	Caprine	8/08	Kebbi	Nigeria
NIG/08-46	Lymph node	Caprine	8/08	Kebbi	Nigeria
NIG/08-47	Lung	Caprine	8/08	Kebbi	Nigeria
NIG/08-48	Lung	Caprine	8/08	Kebbi	Nigeria
NIG/08-49	Spleen	Caprine	8/08	Sokoto	Nigeria
NIG/08-50	Lymph node	Caprine	8/08	Sokoto	Nigeria
NIG/08-51	Lung	Caprine	8/08	Sokoto	Nigeria
NIG/08-52	Lung	Caprine	8/08	Kebbi	Nigeria
NIG/08-53	Spleen	Caprine	8/08	Kebbi	Nigeria
NIG/08-54	Lymph node	Caprine	8/08	Kebbi	Nigeria
NIG/08-55	Spleen	Caprine	8/08	Sokoto	Nigeria
NIG/08-56	Lymph node	Caprine	8/08	Sokoto	Nigeria
CIV/09-OVP	Lung	Ovine	7/09	Fronobo	Côte d’Ivoire
CIV/09-OVG	Lymph node	Ovine	7/09	Fronobo	Côte d’Ivoire
CIV/09-OVF	Liver	Ovine	7/09	Fronobo	Côte d’Ivoire
CIV/09-OVR	Spleen	Ovine	7/09	Fronobo	Côte d’Ivoire
CIV/09-01P	Lung	Caprine	7/09	Amonkro	Côte d’Ivoire
CIV/09-01G	Lymph node	Caprine	7/09	Amonkro	Côte d’Ivoire
CIV/09-01F	Liver	Caprine	7/09	Amonkro	Côte d’Ivoire
CIV/09-01R	Spleen	Caprine	7/09	Amonkro	Côte d’Ivoire
CIV/09-02P	Lung	Caprine	7/09	Amonkro	Côte d’Ivoire
CIV/09-02F	Liver	Caprine	7/09	Amonkro	Côte d’Ivoire
CIV/09-02R	Spleen	Caprine	7/09	Amonkro	Côte d’Ivoire
CIV/09-02N	Kidney	Caprine	7/09	Amonkro	Côte d’Ivoire

**Table 2:** Results of the PPRV isolation trial on cell culture from the pathological specimens that were tested PPRV positive by classical and quantitative RT-PCR. CHS-20, CV1 and Vero were used in this trial.

Tissue	Sample ID	PPRV RT-PCR result on tissue sample	Virus isolation^a^			Detection of PPRV in cell culture supernatant (RT-PCR)		
			CHS-20	CV1^b^	Vero^b^	CHS-20	CV1	Vero
Lung	NIG/08-03	+	3 days	N	N	+	−	−
	NIG/08-17	+	8 days	N	N	+	−	−
	NIG/08-27	+	2 days	N	N	+	−	−
	NIG/08-43	+	2 days	N	N	+	+	+
	CIV/09-OVP	+	N	N	N	−	−	−
	**CIV/09-01P**	+	1 day	N	N	+	+	−c
	CIV/09-02P	+	2 days	N	N	+	−	−

Lymph node	NIG/08-04	+^d^	N	N	N	−	−	−
	NIG/08-10	+^d^	7 days	N	N	+	−	−
	NIG/08-30	+	3 days	N	N	+	−	−
	CIV/09-OVG	+	N	N	N	−	−	−
	**CIV/09-01G**	+	2 days	N	N	+	−c	−

Liver	NIG/08-20	+	N	N	N	−	−	−
	NIG/08-28	+	5 days	N	N	+	−	−
	NIG/08-44	+	8 days	N	N	+	−	−
	CIV/09-OVF	+	N	N	N	−	−	−
	**CIV/09-01F**	+	N	N	N	−	−	−
	CIV/09-02F	+	4 days	N	N	+	−	−

Spleen	NIG/08-11	+^d^	10 days	N	N	+	−	−
	NIG/08-18	+	10 days	N	N	+	−	−
	NIG/08-22	+	8 days	N	N	+	−	−
	NIG/08-29	+	7 days	N	N	+	−	−
	CIV/09-OVR	+	N	N	N	−	−	−
	**CIV/09-01R**	+	N	N	N	−	−	−
	CIV/09-02R	+	N	N	N	−	−	−

Kidney	CIV/09-02N	+	N	N	N	−	−	−

N = no CPE. Samples in bold were used in the RT-PCR shown in Fig. 3.

a Day post infection (dpi) on which the CPE was detected in the cell monolayer.

b Up to 4 blind passages were performed.

c Positive for the RNAs that were extracted from the cells at the time of the first blind passage.

d Positive in real time PCR only.
